# Editorial: In Search of the Neural Underpinnings and a Functional Definition of the Elusive “Working Memory”

**DOI:** 10.3389/fneur.2022.870063

**Published:** 2022-03-24

**Authors:** Dimitrios Kasselimis, Michael Petrides, Juliana Baldo, Constantin Potagas

**Affiliations:** ^1^Neuropsychology and Language Disorders Unit, First Department of Neurology, National and Kapodistrian University of Athens, Athens, Greece; ^2^Cognitive Neuroscience Unit, Montreal Neurological Institute and Hospital, McGill University, Montreal, QC, Canada; ^3^Veterans Affairs Northern California Health Care System, Martinez, CA, United States

**Keywords:** working memory, proactive interference, learning, Hebb repetition paradigm, fMRI, multivariate lesion symptom mapping, primary progressive aphasia, DTI (diffusion tensor imaging)

Working Memory (WM) is one of the most widely used constructs in the fields of Cognitive Psychology, Neuropsychology and Behavioral Neurology. Since the publication of the seminal work by Baddeley and Hitch (1), there has been a plethora of studies introducing psychometric tools for WM assessment, investigating the impact of neurological disorders on WM, and attempting to elucidate its neural underpinnings. However, the specifics of the nature of WM, as well as its underlying mechanisms, in terms of both explanatory cognitive models and identification of the neural networks supporting its individual components, remain elusive. This Research Topic aims to tackle such issues.

Ries et al. examined the consequences of gray and white matter lesions on proactive interference in WM. They recruited 15 stroke patients with unilateral lesions, affecting the left prefrontal cortex, the right prefrontal cortex, and the left temporal cortex, as well as underlying white matter tracts. Their findings indicate an association between the extreme/external capsule fasciculus and proactive interference, and thus highlight the importance of the ventral stream in this aspect of WM. To further strengthen their claim, they present a detailed investigation of a specific patient from their sample, who had relatively spared left inferior frontal gyrus, but severely damaged pathways comprising the ventral stream, and most prominently exhibited a pattern of impaired WM performance.

Loo et al. examined whether cortical regions previously associated with WM processes of auditory stimuli may show evidence of long-term learning, indicated by memory persistence across trials of a Hebb repetition paradigm. The authors implemented representational similarity analysis, in order to assess whether relationships between multivariate patterns of neural activity may be dependent on the task condition (same or different tone sequences). They were particularly interested in the most posterior portion of the left planum temporale, a Sylvian-parietal-temporal region (Spt) and investigated whether this area may show evidence of persistence of sequence-specific representations across trials. Based on their results, which did confirm this association, the authors suggest that Spt, along with other components of the auditory dorsal stream do not seem to function as a buffer, “resetting” after each trial, but rather encode information by forming more persistent memory traces.

Purcell et al. investigated the neural substrate of two specific domains of WM, namely phonological and orthographic. They assessed 37 patients with left hemisphere lesions on measures of phonological and orthographic WM, as well as long-term memory (as a control variable). They used Multivariate Lesion-Symptom Mapping to assess associations between specific lesion sites and distinct WM domains. Their analyses revealed associations between phonological WM performance and significant clusters in the left temporo-parietal cortex, including the supramarginal gyrus, the parietal operculum, and the superior temporal gyrus, while orthographic WM performance was associated with clusters in the left posterior parietal cortex, specifically in the angular gyrus. Overall, their results indicate distinct neural substrates for orthographic and phonological WM, in accordance with a domain-specific buffer account of WM. This is in contrast to the notion of a domain-general WM mechanism supported by the parietal cortex, which previous studies have suggested.

Afthinos et al. investigated the contribution of WM-related brain areas to verbal learning. They assessed 32 patients with Primary Progressive Aphasia using the Rey Auditory Verbal Learning Test. They derived four indices calculated on the basis of immediate, consecutive, and delayed recall scores of the test: Immediate Recall, Verbal Learning, Sum of Learning, and Delayed Recall. They then regressed these indices with perfusion data from pseudo-continuous arterial spin labeling MRI sequences. Their results revealed distinct associations between brain regions and stages of learning. These findings support the notion of differential involvement of WM in the advancing steps of the verbal learning process. The authors conclude that the left angular gyrus, which is thought to support domain-specific (i.e., semantic) WM, is involved in the initial stages of verbal learning, but delayed recall depends solely on temporal areas.

Overall, the articles in this Research Topic cover a wide range of interesting issues related to WM. Notably, the researchers participating in this collection have investigated distinct subcomponents of WM, rather than treating it as a unitary entity. This is of particular importance, since the individual components of WM have not yet been completely clarified, even though there have been studies attempting to elucidate such subcomponents [see, for example, (2, 3)]. It should be further noted that the articles comprising this Topic focus on healthy participants, and also patients from different neurological populations, thus contributing to the ongoing effort of the integration of data derived from lesion and fMRI studies in healthy participants.

In conclusion, this Research Topic offers valuable insights into the neural underpinnings of WM from several different perspectives by integrating neuropsychological and brain imaging methodologies in both healthy participants and neurologic patients.

## Author Contributions

All authors listed have made a substantial, direct, and intellectual contribution to the work and approved it for publication.

## Funding

This research is co-financed by Greece and the European Union (European Social Fund-ESF) through the Operational Programme “Human Resources Development, Education and Lifelong Learning” in the context of the project “Reinforcement of Postdoctoral Researchers – 2nd Cycle” (MIS-5033021), implemented by the State Scholarships Foundation (IKY).

## Conflict of Interest

The authors declare that the research was conducted in the absence of any commercial or financial relationships that could be construed as a potential conflict of interest.

## Publisher's Note

All claims expressed in this article are solely those of the authors and do not necessarily represent those of their affiliated organizations, or those of the publisher, the editors and the reviewers. Any product that may be evaluated in this article, or claim that may be made by its manufacturer, is not guaranteed or endorsed by the publisher.
